# Anti-Inflammatory Effect of Procyanidins from Wild Grape (*Vitis amurensis*) Seeds in LPS-Induced RAW 264.7 Cells

**DOI:** 10.1155/2013/409321

**Published:** 2013-10-23

**Authors:** Min-Ji Bak, Van Long Truong, Hey-Sook Kang, Mira Jun, Woo-Sik Jeong

**Affiliations:** ^1^Department of Food and Life Sciences, College of Biomedical Science & Engineering, Inje University, Gimhae 621-749, Republic of Korea; ^2^Department of Food Science and Nutrition, Dong-A University, Busan 604-714, Republic of Korea

## Abstract

In the present study, the anti-inflammatory effect and underlying mechanisms of wild grape seeds procyanidins (WGP) were examined using lipopolysaccharide- (LPS-) stimulated RAW 264.7 cells. We used nitric oxide (NO) and prostaglandin E_2_ (PGE_2_) and reactive oxygen species (ROS) assays to examine inhibitory effect of WGP and further investigated the mechanisms of WGP suppressed LPS-mediated genes and upstream expression by Western blot and confocal microscopy analysis. Our data indicate that WGP significantly reduced NO, PGE_2_, and ROS production and also inhibited the expression of proinflammatory mediators such as inducible nitric oxide synthase (iNOS) and cyclooxygenase-2 (COX-2) protein expressions. Consistently, WGP significantly reduced LPS-stimulated expression of proinflammatory cytokines such as tumor necrosis factor **α** (TNF-**α**) and interleukin- (IL-) 1**β**. Moreover, WGP prevented nuclear translocation of nuclear factor-**κ**B (NF**κ**B) p65 subunit by reducing inhibitory **κ**B-**α** (I**κ**B**α**) and NF**κ**B phosphorylation. Furthermore, we found that WGP inhibited LPS-induced phosphorylation of p38 mitogen-activated protein kinase (MAPK). Taken together, our results demonstrated that WGP exerts potent anti-inflammatory activity through the inhibition of iNOS and COX-2 by regulating NF**κ**B and p38 MAPK pathway.

## 1. Introduction

*Vitis amurensis*, a wild-growing grape species, is widely distributed in Republic of Korea, China, and Japan. The root and stem have been used as traditional medicines for treatment of cancer and various pains in Republic of Korea and Japan [1]. The fruit is not consumed fresh but is used primarily for production of juice and wine due to its strong stringency. Some studies have suggested that wild grape root and stem have antiangiogenic [2], antioxidant [3], anti-inflammatory [4], and neuroprotective effects [5]. In a recent study, our group addressed the separation and chemopreventive properties of procyanidins from wild grape seeds relating their induction of nuclear factor E2-related factor 2 (Nrf2)/antioxidant response element (ARE) pathway in the human hepatoma HepG2 cell line [6]. Some procyanidins from other species have been found to display anti-inflammatory effect [7, 8], but their molecular mechanisms underlying the anti-inflammatory effects have not been elucidated. 

 Inflammation is involved in a variety of chronic diseases including cancer and heart disease. Pathogen- and host-derived molecules, such as lipopolysaccharide (LPS) and interferon-*γ* (IFN-*γ*), stimulate macrophages to, in turn, upregulate inflammatory mediators such as nitric oxide (NO), prostaglandin E_2_ (PGE_2_), and reactive oxygen species (ROS), as well as proinflammatory mediators such as inducible nitric oxide synthase (iNOS) and cyclooxygenase-2 (COX-2) [9]. NO is overproduced endogenously by iNOS which is induced in response to proinflammatory cytokines and LPS [10]. COX-2 is also induced by several stimuli and is responsible for the production of large amounts of proinflammatory prostaglandins at the inflammatory sites [11]. Therefore, the inhibition of these inflammation mediators is an important target pathway in the treatment of disease with anti-inflammatory components [12, 13]. 

 Multiple studies have shown that the expressions of several cytokines genes, including tumor necrosis factor-*α* (TNF-*α*) and interleukin 1*β* (IL-1*β*), are associated with activation of nuclear factor-*κ*B (NF*κ*B), which is a transcription factor that plays a major role in the regulation of genes associated with inflammation [14–16]. In non-stimulated cells, NF*κ*B dimmers of p50 and p65 subunits remain inactive in the cytoplasm through interaction with an inhibitory protein, I*κ*B. However, in response to cell stimulation, I*κ*B kinase complex is rapidly degraded and phosphorylated. The transcription factor, NF*κ*B, then translocates into the nucleus and binds to the DNA site that regulates transcription of inflammatory mediators [17]. Recently, many studies have demonstrated the role of phytochemicals in anti-inflammatory activity through downregulation of NF*κ*B pathway [18–20]. 

 The mitogen-activated protein kinases (MAPKs) such as extracellular signal regulated kinase (ERK), p38, and c-Jin NH2-terminal kinase (JNK) are a group of signaling molecules that play a critical role in the regulation of cell growth and differentiation, as well as in the control of cellular responses to cytokines and stresses [21, 22]. Phosphorylation of MAPKs is known to be a critical component in the production of NO and proinflammatory cytokines in activated macrophages [23, 24]. Also, it has been demonstrated that the specific MAPK inhibitors suppress the expression of the iNOS and COX-2 genes [6, 25]. Moreover, several studies have shown that MAPKs play a critical role in the activation of NF*κ*B [23, 26]. In addition, it has been shown that the PI3K/Akt signaling pathway plays an important role in negatively regulating LPS-induced acute inflammatory responses *in vitro* and *in vivo* [27, 28]. Inhibition of the PI3K/Akt signaling pathway can enhance the activation of NF*κ*B transcription factors and the expression of iNOS and COX-2 in RAW 264.7 cells [29]. 

 In this present study, we examine the anti-inflammatory effects of wild grape seed procyanidins (WGP) in LPS-stimulated RAW 264.7 cells. The results show that WGP suppressed LPS-induced NO, PGE_2_ and ROS production by inhibiting activation of NF*κ*B pathway, as well as the p38 MAPK signaling pathway in LPS-stimulated RAW 264.7 cells.

## 2. Materials and Methods

### 2.1. Materials

Lipopolysaccharide (LPS, *Escherichia coli* O127:B8) was obtained from Sigma-Aldrich Co. (St. Louis, MO). 2′,7′-Dichlorodihydrofluorescein (DCF-DA) was purchased from Molecular Probes Inc. (Eugene, OR). Dulbecco's modified Eagle's medium, fetal bovine serum (FBS), and penicillin/streptomycin were obtained from Hyclone (Logan, UT). Antibodies against iNOS, COX-2, and *β*-actin, as well as horseradish peroxidase-conjugated anti-goat and anti-rabbit IgG, were purchased from Santa Cruz Biotech (Santa Cruz, CA). Anti-phospho-JNK, phospho-ERK, phospho-p38, phospho-I*κ*B*α*, phospho-p65, I*κ*B*α*, p65, IL-1*β*, and TNF-*α* were purchased from Cell Signaling Technology Inc. (Beverly, MA). All other reagents used in this study were of the highest grade available.

### 2.2. Preparation of WGP

Seeds were collected from wild gape (*Vitis amurensis*) from a vineyard (Dooraemaeul, Inc., Hamyang, Republic of Korea), during transfer of the musts for wine fermentation. WGP were extracted from wild grape seeds and analysed as described previously [6]. Briefly, dried powder of wild grape seeds were extracted with 70% aqueous acetone. After the extraction, 70% acetone extract of wild grape seeds was partitioned with *n*-hexane to remove hydrophobic compounds and chromatographed over a Toyopearl HW-400F (Tosho, Tokyo, Japan) using an aqueous solution of 50% MeOH and 66% acetone and 100% acetone to yield a procyanidins fraction. The procyanidins fraction was analysed with various analytical techniques including the vanillin assay, butanol-HCl hydrolysis, and HPLC-MS analysis after depolymerization with phloroglucinol. The major procyanidins of WGP were determined as a mixture of prodelphinidins and procyanidins with the average polymerization degree of 6.22 and 4.65, respectively.

### 2.3. Cell Culture

RAW 264.7 cells were purchased from American Type Cell Culture (ATCC, Rockville, MD) and maintained in Dulbecco's modified Eagle's medium (DMEM) supplemented with 10% fetal bovine serum and 100 U/mL of penicillin/streptomycin and maintained at 37°C in a humidified CO_2_ incubator.

### 2.4. Cell Viability Assay

Cell viability was determined by the CellTiter 96 Non-Radioactive Cell Proliferation Assay (Promega Corp., Madison, WI) according to the manufacturer's instructions. Briefly, cells were plated at a density of 2 × 10^4^ cells in a 24-well plate, and WGP was added to each plate at the indicated concentrations. After 24 h incubation period, the absorbance was measured at 490 nm with a PowerWave XS microplate reader (BioTek Instruments, Inc., Winooski, VT). This assay was repeated three times with triplicate samples at each measurement. 

### 2.5. Measurement of ROS Production

The level of intracellular ROS was quantified by fluorescence with DCF-DA. The cells (2 × 10^4^ cells/well) were plated in 96 well plates and preincubated with the DCF-DA for 1 h at 37°C in darkness. After washing out the excess probe, the cells were treated with WGP or WGP and LPS for 24 h. The fluorescence was measured at 485/20 nm excitation and 528/20 nm emission in a fluorescence multidetection reader (Synergy HT Multidetection Microplate Reader; BioTek, VT).

### 2.6. Measurement of NO Production

The RAW 264.7 cells were plated at 1 × 10^5^ cells/well in 24 well plates and then incubated with or without LPS (1 *μ*g/mL) in the absence or presence of WGP for 24 h. Nitrite levels in culture media were determined using the Griess reaction assay and presumed to reflect NO levels. Briefly, 100 *μ*L of cell culture medium was mixed with 100 *μ*L of Griess reagent (equal volumes of 1% (w/v) sulfanilamide in 5% (v/v) phosphoric acid, and 0.1% (w/v) naphthyl ethylenediamine dihydrochloride), incubated at room temperature for 10 min and then the absorbance at 540 nm was measured in a microplate reader (PowerWave XS). The amount of nitrite in the samples was measured with the serial dilution standard curve of sodium nitrite.

### 2.7. Measurement of PGE_2_ Production

The amount of PGE_2_ produced from endogenous arachidonic acid was measured using a PGE_2_ Parameter Assay Kit (R&D Systems, Minneapolis, MN). RAW 264.7 cells (1 × 10^5^ cells/well) were treated with WGP for 1 h and stimulated with LPS for 24 hr, the conditioned media was collected to perform PGE_2_ enzyme immune-metric assay according to the manufacturer's protocol.

### 2.8. Preparation of Whole Cell, Cytosolic, and Nuclear Extracts

The preparation of whole cell extract was previously described [6]. RAW 264.7 cells were seeded at 2 × 10^5^ cells/well on 6-well plates and were treated with WGP and stimulated with LPS. After treatment, cells were collected by centrifugation and washed twice with ice-cold phosphate buffered saline. The cells pellets were resuspended in lysis buffer on ice for 1 h; and cell debris was removed by centrifugation. The cytosolic and nuclear proteins were extracted using a Nuclear and Cytoplasmic Extraction Reagents kit (Pierce Biotechnology Inc., Rockford, IL), and protein levels were determined by BCA protein assay (Pierce biotechnology).

### 2.9. Western Blot Analysis

Equal amounts of proteins (whole cell extracts: 30 *μ*g/lane, cytosolic extracts: 30 *μ*g/lane, nuclear extracts: 10 *μ*g/lane) were loaded onto a 12% SDS-polyacrylamide gel electrophoresis unit and then transferred onto a PVDF membrane (Bio-Rad, Hercules, CA). The membranes were incubated in a blocking buffer (5% w/v skim milk in PBST) for 1 h, and then incubated with overnight with primary antibody. After washing three times with 0.1% PBST buffer, the membranes were incubated with the anti-goat or anti-rabbit secondary antibodies conjugated with horseradish peroxidase and detected by the Western Blotting Luminol Reagent (Santa Cruz Biotechnology).

### 2.10. Confocal Microscopy Analysis

Cells were plated at 2 × 10^5^ cells/well on cover glasses bottom dishes and fixed with 4% paraformaldehyde in phosphate-buffered saline for 10 min at RT. Permeabilization was performed in PBS with 0.3% Triton X-100 for 10 min at RT. After blocking for 2 h with 3% bovine serum albumin, the cells were incubated with anti-p65 primary antibody at RT for 2 h. After washing with PBS, Alexa Fluor 555-confugated secondary antibody (Cell signaling) was added for 2 h in the dark. Nuclei were stained with 4′,6′-diamidino-2-phenylindole (DAPI; Thermo Scientific, Rockford, IL), and the cells were visualized under LSM 510 laser confocal microscope (Zeiss, Jena, Germany).

### 2.11. Statistical Analysis

The data were expressed as means ± standard deviation (S.D.). Statistical analyses were performed using SigmaPlot 8.0 software (Systat Software Inc., Chicago, IL). Student's *t*-test and one-way ANOVA were used to determine the statistical significant difference between the LPS-treated and WGP plus LPS-treated cells. *P* value of <0.05 was considered statistically significant.

## 3. Results 

### 3.1. Effects of WGP on Cell Viability in LPS-Induced RAW 264.7 Cells

**Figure 1:**
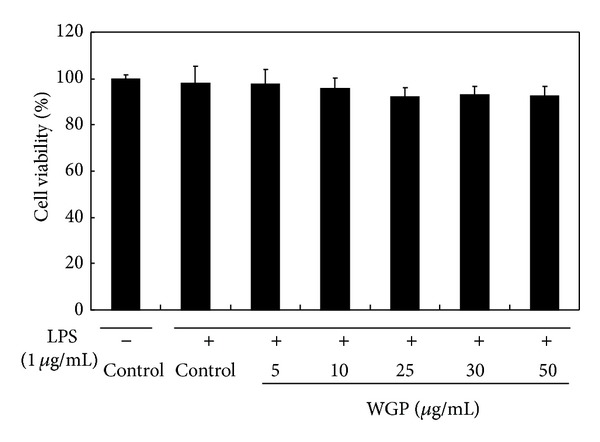
Effects of WGP on cell viability in RAW 264.7 cells. Cells were treated with the indicated concentration of WGP in combination with LPS for 24 h. Cell viabilities were assessed using 3-(4,5-dimethylthiazol-2-yl)-2,5-diphenyltetrazolium  bromide (MTT) assay. Each value represents means ± SD of six independent experiments.

 The effects of WGP on the viability of RAW 264.7 cells were determined by a colorimetric MTS assay after 48 hr treatments. The data were expressed as percent cell viability compared to those of control (DMSO, 0.1%) (Figure 1). WGP did not cause any cytotoxicity at 50 *μ*g/mL in RAW 264.7 cells. Therefore, subsequent experiments were performed with concentrations at or below 50 *μ*g/mL.

### 3.2. Effects of WGP on the Production of ROS in LPS-Induced RAW 264.7 Cells

**Figure 2:**
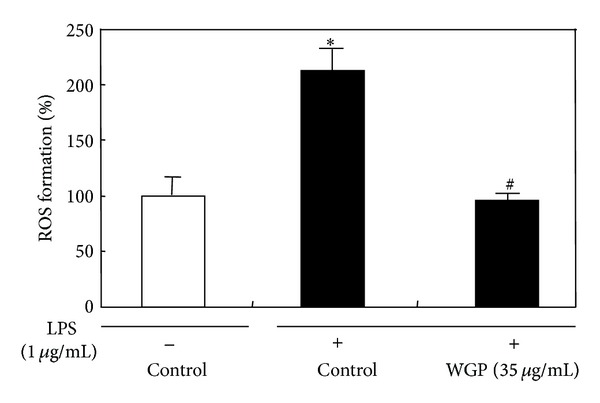
Effects of WGP on LPS-induced ROS production in RAW 264.7 cells. The level of intracellular ROS was measured with DCF-DA. The cells were pretreated with DCF-DA for 1 h, and then exposed to WGP at 35 *μ*g/mL for 24 h. The formation of ROS in the cells was evaluated by the arbitrary fluorescence unit and described as fold induction test via vehicle (DMSO, 0.1%). Each value represents mean ± SD of six independent experiments. *P < 0.05 indicates differences from the unstimulated control group. ^#^ *P* < 0.05 indicates differences from the LPS-treated group.

We examined the effects of WGP on LPS-induced ROS production in RAW 264.7 cells. Treatment of RAW 264.7 cells with LPS rapidly increased intracellular ROS level, as determined by using DCF-DA, which was effectively attenuated by pretreatment with WGP (Figure 2).

### 3.3. Effects of WGP on the Production of NO and PGE_2_ in LPS-Induced RAW 264.7 Cells

**Figure 3:**
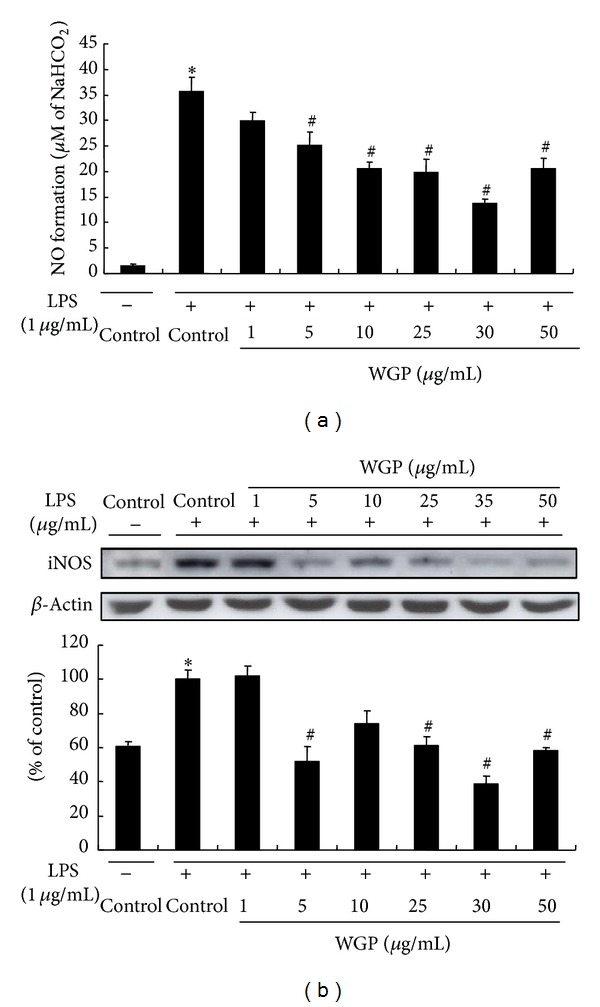
Effects of WGP on LPS-induced NO and iNOS protein expression in RAW 264.7 cells. (a) NO formation. The cells were treated with LPS only or with WGP ranging from 1 to 50 *μ*g/mL for 24 h. The culture media were collected, and the nitric oxide concentration was measured by the Griess reaction. Each value represents mean ± S.D. of triplicate experiments. (b) The cells were treated with WGP ranging from 1 to 50 *μ*g/mL for 1 h and then treated with LPS for 24 h. Equal amounts of total protein were subjected to Western blot analysis as described in Section 2. The ratio of immunointensity between the iNOS and the *β*-actin was calculated. The bar represents means ± S.D. from three independent experiments. *P < 0.05 indicates differences from the unstimulated control group. ^#^ *P* < 0.05 indicates differences from the LPS-treated group.

**Figure 4:**
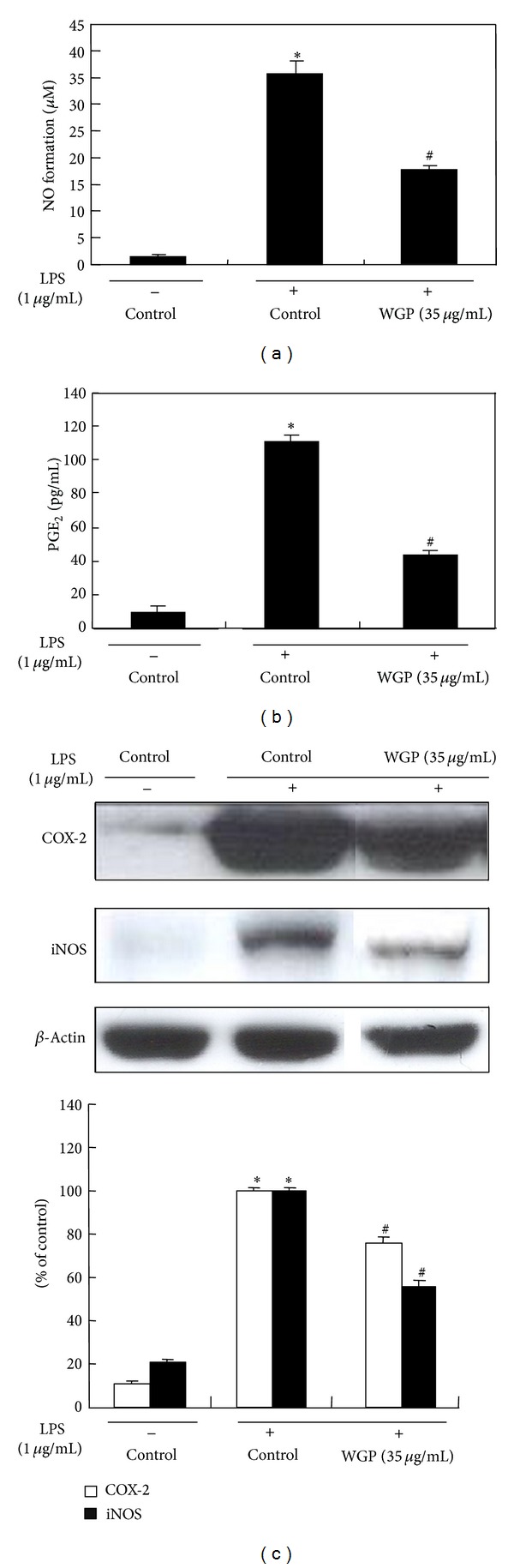
Effects of WGP on LPS-induced NO and PGE_2_ production and iNOS and COX-2 protein expression in RAW 264.7 cells. (a) NO formation. The cells were treated with LPS only or with WGP at 35 *μ*g/mL for 24 h. The culture media were collected, and the nitric oxide concentration was measured by the Griess reaction. (b) PGE_2_ formation. Each culture supernatant was collected, and the amount of PGE_2_ was measured using the PGE_2_ parameter assay kit. Each value represents mean ± SD of triplicate experiments. (c) The cells were treated with WGP (35 *μ*g/mL) for 1 h and then treated with LPS for 24 h. Equal amounts of total protein were subjected to Western blot analysis as described in Section 2. The ratio of immunointensity between the iNOS/COX-2 and the *β*-actin was calculated. Each bar (open bar, iNOS; closed bar, COX-2) represents means ± S.D. from three independent experiments. *P < 0.05 indicates differences from the unstimulated control group. ^#^ *P* < 0.05 indicates differences from the LPS-treated group.

The effects of WGP on the level of NO and PGE_2_ in the culture media of RAW 264.7 cells were determined after 24 h treatment of 1 *μ*g/mL LPS with WGP. NO production and iNOS protein expression by WGP were evaluated with the dose range of 1–50 *μ*g/mL. Treatment with WGP at 35 *μ*g/mL dramatically inhibited the NO production and iNOS protein expression in LPS-stimulated RAW 264.7 cells (Figure 3). Therefore, further experiments were performed with concentrations at 35 *μ*g/mL. As illustrated in Figure 4(a), treatment of the cells with LPS resulted in increased NO production; however, WGP at 35 *μ*g/mL significantly inhibited the production of NO. Treatment with WGP also significantly decreased LPS-induced PGE_2_ production (Figure 4(b)).

### 3.4. Effects of WGP on the Protein Expression of iNOS and COX-2 in LPS-Induced RAW 264.7 Cells

To investigate whether the inhibitory effect of WGP on NO and PGE_2_ production was via inhibition of corresponding gene expression, the protein expressions of iNOS and COX-2 were determined by Western blot. As displayed in Figure 4(c), the protein levels of iNOS and COX-2 were undetectable in RAW 264.7 cells without LPS stimulation. Treatment with LPS alone markedly increased iNOS and COX-2 protein levels, whereas cotreatment with WGP significantly suppressed the expression of iNOS and COX-2 proteins. The reduced expressions of iNOS and COX-2 protein were consistent with the reductions in total NO and PGE_2_ in culture media. 

### 3.5. Effects of WGP on the Protein Expression of TNF-*α* and IL-1*β* in LPS-Induced RAW 264.7 Cells

**Figure 5:**
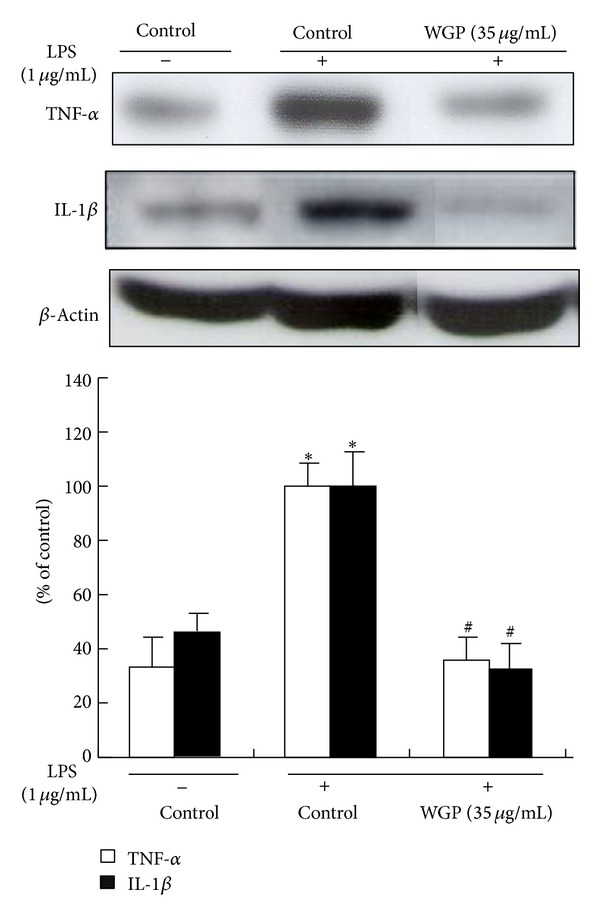
Effects of WGP on LPS-induced TNF-*α* and IL-1*β* protein expression in RAW 264.7 cells. The cells were treated with WGP (35 *μ*g/mL) for 1 h and then treated with LPS for 24 h. Equal amounts of total protein were subjected to Western blot analysis, as described in Section 2. The ratio of immunointensity between the TNF-*α*/IL-1*β* and the *β*-actin was calculated. Each bar (open bar, TNF-*α*; closed bar, IL-1*β*) represents means ± SD from three independent experiments. *P < 0.05 indicates differences from the unstimulated control group. ^#^ *P* < 0.05 indicates differences from the LPS-treated group.

To examine the effects of WGP on the expression of proinflammatory cytokines following LPS treatment, Western blot analyses were performed. As shown in Figure 5, LPS significantly stimulated the expression of TNF-*α* and IL-1*β*. On the contrary, WGP significantly inhibited the LPS-stimulated TNF-*α* and IL-1*β* expression. 

### 3.6. Effects of WGP on LPS-Induced Nuclear Translocation of NF*κ*B and on the Phosphorylation of I*κ*B*α* in LPS-Induced RAW 264.7 Cells

**Figure 6:**
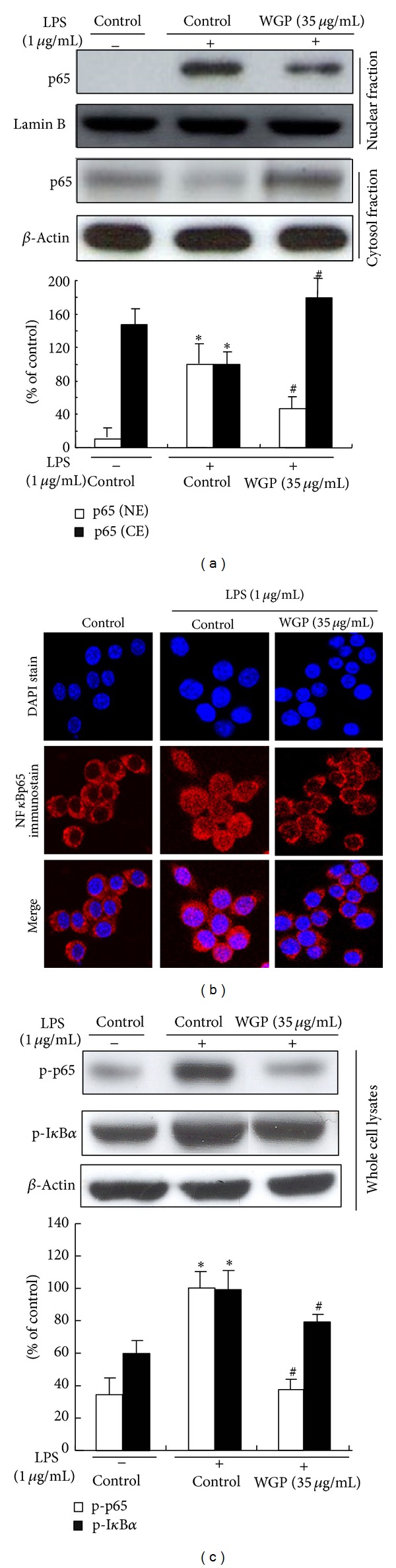
Effects of WGP on LPS-induced p65 nuclear translocation and p65 and I*κ*B*α* phosphorylation in RAW 264.7 cells. (a) The cells were treated with 1 *μ*g/mL LPS alone or with WGP (35 *μ*g/mL) for 2 h. Cytosolic and nuclear fractions were prepared and analyzed by Western blotting. The ratio of immunointensity between the p65 (nuclear fraction: NE)/p65 (cytosolic fraction: CE) and the Lamin B and *β*-actin was calculated. Each bar (open bar, p65 (NE); closed bar, p65 (CE)) represents means ± S.D. from three independent experiments. (b) Cells were pretreated with WGP (35 *μ*g/mL) for 1 h followed by stimulation with LPS for 2 h. Samples were stained by anti-p65 antibody and DAPI then prepared for confocal microscopy analysis. (c) The whole cells were treated with WGP at 35 *μ*g/mL for 2 h and treated with LPS for 1 h. Equal amounts of total protein were subjected to Western blot analysis. The ratio of immunointensity between the p-p65/p-I*κ*B*α* and the *β*-actin was calculated. Each bar (open bar, p-p65; closed bar, p-I*κ*B*α*) represents means ± S.D. from three independent experiments. *P < 0.05 indicates differences from the unstimulated control group. ^#^ *P* < 0.05 indicates differences from the LPS-treated group.

Because levels of iNOS and COX-2 protein were inhibited by WGP, we then examined the effects of WGP on the activation of NF*κ*B. The translocation of NF*κ*B was measured by extracts of nucleus and cytosol and subjected to analyses of Western blot and immunofluorescence. LPS stimulation for 1 h caused the translocation of p65, a subunit of NF*κ*B, to the nucleus in the macrophage cells (Figure 6(a)). However, WGP treatment effectively blocked the LPS-induced nuclear translocation of p65 in the cells. These results were confirmed by NF*κ*B and DAPI costaining in LPS-treated RAW 264.7 cells (Figure 6(b)). As illustrated in Figure 6(c), the treatment with WGP attenuated the LPS-stimulated phosphorylation of p65 as well as that of I*κ*B*α*.

### 3.7. Effects of WGP on MAPKs and Akt Phosphorylation in LPS-Induced RAW 264.7 Cells

**Figure 7:**
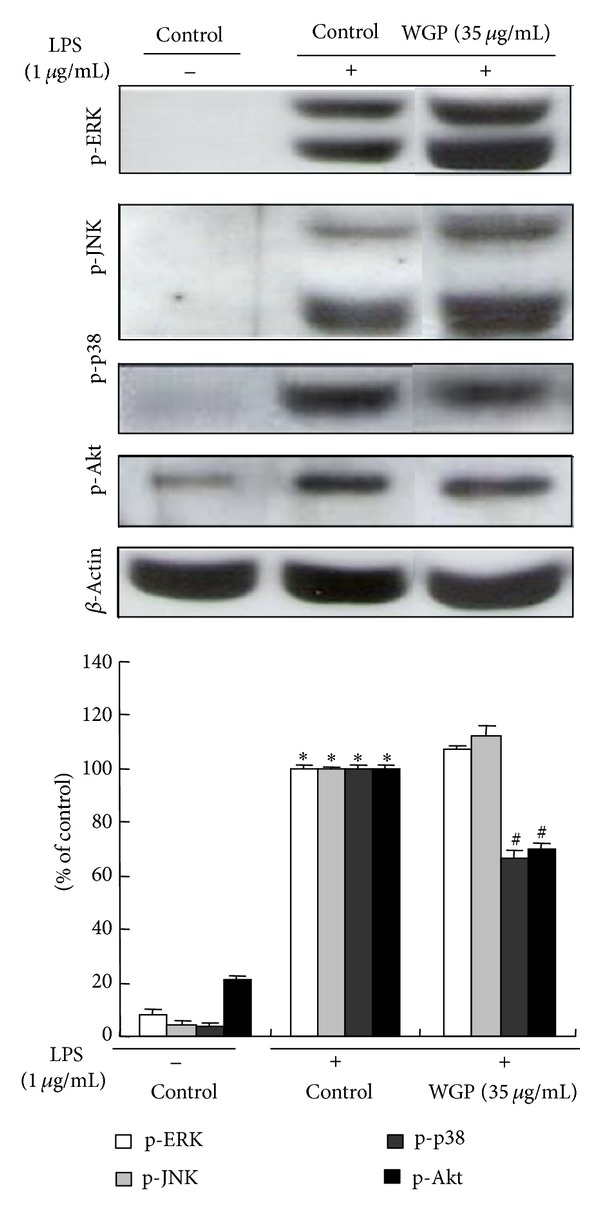
Effects of WGP on LPS-induced activation of MAP kinases and Akt in RAW 264.7 cells. The expression of phospho-ERK 1/2 (p-ERK), phospho-p38 (p-p38), phospho-JNK 1/2 (p-JNK), and phospho-Akt (p-Akt) was analyzed by Western blot. The cells were treated with WGP at 35 *μ*g/mL for 1 h and treated with LPS (1 *μ*g/mL) for 1 h. *P < 0.05 indicates differences from the unstimulated control group. ^#^ *P* < 0.05 indicates differences from the LPS-treated group.

To investigate whether the inhibition of inflammatory response by WGP is mediated through the MAPK and PI3K/Akt pathways, we examined the effect of WGP on the LPS-stimulated phosphorylation of upstream kinases including ERK1/2, JNK, p38, and Akt in RAW 264.7 cells. As displayed in Figure 7, WGP significantly inhibited the phosphorylation of p38 and its possible upstream kinase Akt, whereas phosphorylation of ERK and JNK was not affected. These results suggest that the anti-inflammatory effect of WGP might come from its modulation on p38/Akt signaling pathway.

## 4. Discussion

Procyanidins, plant polyphenols formed by the polymerization of flavan-3-ols, as cytoprotective agents have become an important source in human health research [30]. Epidemiological studies have indicated that populations that consume procyanidin-rich foods have lower incidences of inflammatory diseases [31]. Our previous study successfully demonstrated the separation and chemopreventive properties of procyanidins from wild grape seeds [6]. In this study, we investigated the anti-inflammatory activities of procyanidins, a main component isolated from wild grape (*Vitis amurensis*) seeds, in LPS-stimulated RAW 264.7 cells.

 Macrophages are generally an important component in the immune defense mechanism. During the progress of inflammation, macrophages actively participate in inflammatory responses by releasing proinflammatory cytokines and mediators [32]. Furthermore, proinflammatory mediators such as ROS, NO, iNOS, and COX-2 play a key role in the pathogenesis of many acute and chronic inflammatory diseases [33]. ROS are well documented to function as signaling molecules, stimulating cellular activities ranging from cytokine secretion to cell proliferation, and at higher concentration, they can induce cell injury and death [34]. The iNOS and COX-2 pathway is known to play an important role in inducing ROS production [35, 36]. Since ROS is critical for LPS-induced inflammation through the activation of NF*κ*B-related signaling [37], we first performed experiments to determine the effects of WGP on intracellular ROS accumulation. Pretreatment of cells with WGP significantly reduced the LPS-induced ROS production. Recent studies also demonstrated that lipid soluble extracts of red ginseng and *Salvia miltiorrhiza* possessed anti-inflammatory effects in LPS-induced RAW 264.7 cells by decreasing ROS production [38, 39]. Overproduction of NO produced by overexpression of iNOS has been implicated in the pathogenesis of septic shock, inflammation, and carcinogenesis [40]. COX-2 is another inducible enzyme that catalyzes biosynthesis of PGE_2_, which contributes to pathogenesis of various inflammatory diseases, edema, angiogenesis, invasion, and growth of tumor [41]. Recently, mounting evidence both *in vitro* and *in vivo* has indicated an existing cross-talk between the release of NO and PGE_2_ in the modulation of molecular mechanisms that regulate inflammation pathway [42, 43]. Thus, the anti-inflammatory agents that decrease NO and PGs production by simultaneously inhibiting the iNOS and COX-2 gene may have a potential therapeutic effect on the treatment of inflammatory and infectious diseases. According to our results, WGP strongly inhibits LPS-induced NO and PGE_2_ production by attenuating the protein expression of iNOS and COX-2 without notable cytotoxicity. Our data implicate that WGP might inhibit NO and PGE_2_ production by regulating the transcription molecules of iNOS and COX-2, which could be activated by LPS treatment. Several plant-derived components including curcumin, resveratrol, isoflavones, and red ginseng oil, have been reported to inhibit iNOS and COX-2 and exert anti-inflammatory activities in different types of cells [39, 44–46]. 

 Inflammatory disorders are characterized among other events, by the production of significant amounts of free radicals and nitrogen reactive species as well as cytokines such as TNF-*α*, IL-1*β*, and IL-6 [47]. In particular, IL-1*β* is an important component in the initiation and enhancement of inflammatory response. TNF-*α* is also a pivotal proinflammatory cytokine and is regarded as an endogenous mediator of LPS-induced fever [48]. Our results showed that WGP could significantly suppress TNF-*α* and IL-1*β* expression. Excessive production of cytokines can be induced by inflammatory stimuli such as LPS treatment in macrophages and it will increase the immune response which in turn results in inflammation [49]. Therefore, the inhibition of the proinflammatory cytokines has been identified as a target for anti-inflammatory therapies, supporting our findings that WGP possesses an anti-inflammatory activity via the inhibition of TNF-*α* and IL-1*β* protein expression. 

 Much evidence suggests that transcription factor NF*κ*B is involved in the regulation of LPS-induced inflammatory gene expression [17, 50]. Among the promoter regions of iNOS and COX-2, the transcription factor NF*κ*B binding site is mainly responsible for the transcription activation of these genes by LPS stimulation [51]. Our results demonstrate that WGP has ability to inhibit LPS-induced phosphorylation of p65 and I*κ*B*α* as well as the nuclear translocation of p65. This inhibitory effect might be through suppressing the phosphorylation and proteasome-mediated degradation of its inhibitor I*κ*B*α*. In addition, phosphorylation of NF*κ*B by upstream kinase has been reported to increase transcriptional potential of p65 subunit in LPS-stimulated macrophage [52]. The therapeutic potential of inhibiting NF*κ*B pathway in chronic inflammatory diseases and inflammatory bowel disease has also been reported [53]. Moreover, recent studies have suggested that several natural products suppress inflammatory responses by regulating the NF*κ*B pathway [12, 54]. These findings concur with our finding that the transcriptional inhibition of proinflammatory mediators by WGP is associated with the blockade of NF*κ*B signaling pathway. 

**Figure 8:**
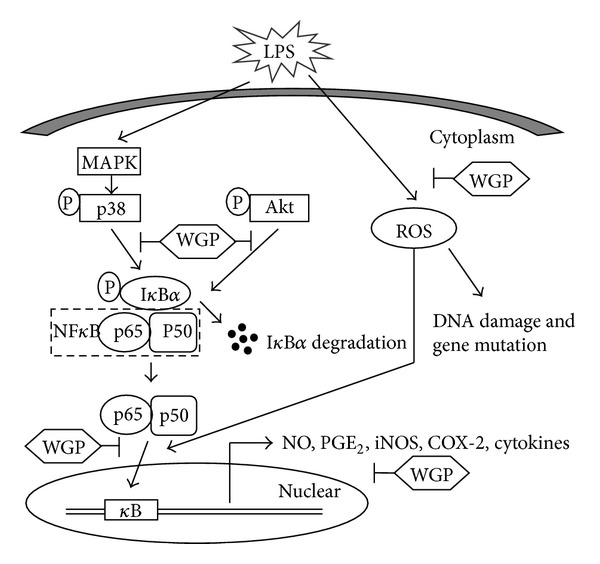
Schematic diagram illustrating the signaling pathways involved in WGP's anti-inflammatory effects in LPS-induced RAW 264.7 cells.

 In addition to NF*κ*B, LPS is a potent activator of MAPK and PI3K/Akt pathways. MAPKs not only play an important role in the LPS-mediated expression of iNOS and COX-2 but also regulate cytokine release in RAW 264.7 cells [26]. In this study, WGP treatment markedly suppressed LPS-stimulated phosphorylation of p38 and Akt, suggesting that suppression of p38 MAPK phosphorylation by WGP might be involved in the inhibition of LPS-induced production of proinflammatory substances in RAW 264.7 cells. Other studies have reported that NF*κ*B-dependent gene expression is downregulated by p38 pathway or by dominant-negative p38 expression, but no significant difference was observed in NF*κ*B translocation and DNA binding, which suggests that p38 could have an indirect influence on NF*κ*B transcription [55, 56]. Additionally, the present result showed that WGP inhibits the LPS-induced phosphorylation of Akt, which is a critical step in PI3K activation. The PI3K/Akt pathway has also been shown to control a variety of cellular processes, including cell survival and proliferation [57]. Recently, studies have shown that the PI3K/Akt signaling pathway plays a crucial role in regulating LPS-induced acute inflammatory responses *in vitro* and *in vivo* [58–60]. However, the role of PI3K/Akt signaling cascades in the regulation of NF*κ*B transactivation remains controversial. The present study agrees with previous investigations which demonstrate that PI3K/Akt pathway promotes the p65 inhibition [61]. However, other studies showed that the inhibition of the PI3K/Akt pathway augmented the p65 activation [59]. The reason for such inconsistency is not clear at this moment and further studies are needed to elucidate the exact molecular mechanisms involved in anti-inflammation by different agents. These results suggest that WGP may block LPS-induced NF*κ*B translocation by inhibiting the phosphorylation MAPKs and PI3K/Akt, and subsequently decreasing the NO, PGE_2_, and ROS production and the protein levels of iNOS, COX-2, and cytokines (Figure 8). 

## 5. Conclusion

In conclusion, our findings indicate that WGP was shown to suppress many inflammatory events including production of NO, PGE_2_, and ROS in RAW 264.7 cells stimulated with LPS. In addition, WGP plays a role in suppressing the protein expressions of iNOS and COX-2, two critical inducible enzymes responsible for the production of NO and PGE_2_, as well as the expression of proinflammation cytokines such as TNF-*α* and IL-1*β*. These effects might be mediated through the inhibition of NF*κ*B activity via downregulation of the p38 MAPK and Akt signaling pathways. Taken together, WGP may be used as a potent natural anti-inflammatory agent. 

## Abbreviation

WGP: Wild grape seeds procyanidins
LPS: Lipopolysaccharide
NO: Nitric oxide
PGE_2_: Prostaglandin E_2_
ROS: Reactive oxygen species
iNOS: Inducible nitric oxide synthase
COX-2: Cyclooxygenase 2
TNF-*α*: Tumor necrosis factor *α*
I*κ*B*α*: Inhibitory *κ*B-*α*
NF*κ*B: Nuclear factor-*κ*B
ERK: Extracellular regulated kinase
JNK: c-Jun N-terminal kinase
PI3K: Phosphoinositide 3-kinase
MAPK: Mitogen-activated protein kinase
DCF-DA: 2′,7′-Dichlorodihydrofluorescein.
